# The Differential Association Between Leadership Styles and Organizational Silence in a Sample of Chinese Nurses: A Multi-Indicator and Multicause Study

**DOI:** 10.1155/jonm/9626175

**Published:** 2025-04-03

**Authors:** Guangling Hu, Zhiyang Guo, Yu Wang, Luwen Wang

**Affiliations:** ^1^Department of Cardiovascular Medicine, Fuwai Central China Cardiovascular Hospital, Zhengzhou, Henan, China; ^2^School of Nursing, Xinxiang Medical University, Xinxiang, Henan, China; ^3^Department of Nursing, Fuwai Central China Cardiovascular Hospital, Zhengzhou, Henan, China; ^4^Heart Failure Wards, Fuwai Central China Cardiovascular Hospital, Zhengzhou, Henan, China

**Keywords:** Chinese nurse, differential association, leadership style, multiple indicators, organizational silence

## Abstract

**Objective:** To determine the effect of diverse leadership styles on the organizational silence of nurses and the association between demographic factors and organizational silence of nurses.

**Background:** Organizational silence prevails among nurses, threatening patient safety and hospital innovation. Transformational and transactional leadership negatively affect nurses' organizational silence, but further confirmation is needed.

**Methods:** The 545 clinical nurses from four hospitals in Shenzhen completed the online self-report questionnaires including multivariate leadership style scale and nurses' organizational silence assessment questionnaire.The data were analyzed by SPSS26.0 software using analysis of variance and Pearson's correlation. The Multiple Indicators Multiple Causes (MICIC) model was used to analyze the influencing factors of organizational silence using AMOS 24.

**Results:** The results of the univariate analysis revealed that the differences in the organizational silence scores of the nurses based on demographic factors such as age, gender, professional title, and undertaking nursing management tasks were not significant (all *p* > 0.05). However, the difference in the employment status was significant (*p* < 0.05). The MICIC model showed that the transformational leadership can aggravate this organizational silence of the nurses (*β* = 0.59, *p* < 0.001), whereas transactional leadership style and employment status had no significant effects (both *p* > 0.05).

**Conclusion:** The organizational silence of nurses was at a moderately low level and transformational leadership style contributes to organizational silence of the nurses. The findings of this study suggested that nursing managers should strive for authentic and open leadership, pay attention to individual differences, and adjust their leadership style according to the preferences and needs of nurses, achieving personalized adaptation of leadership.

## 1. Introduction

Nursing strongly influences the overall delivery of medical care, and the quality of nursing directly affects patient outcomes. Although nurses are often at the forefront of clinical issues, sometimes they hesitate to voice their concerns, organizational management issues, patient safety, and personal emotional events [1] to nursing managers or seniors. While nurses can share their perspectives based on their extensive experience and knowledge, they may not share their views for various personal, organizational, and managerial reasons. For instance, in a hierarchical nursing organization, new nurses may initially remain silent to adapt to the specific organizational culture and unwritten rules, only beginning to fulfill their nursing functions after becoming accustomed to this environment [2]. This phenomenon, known as organizational silence among nurses, is pervasive among clinical nurses. Nurses exhibit moderate levels of organizational silence as shown in a recent study in China [3], a finding that is corroborated by studies in other countries [4]. For example, a survey [5]showed that 91.2% of nurses experienced organizational silence and 61.6% said they would choose to remain silent when confronted with a major problem.

Organizational silence can be divided into four categories. Acquiescent silence mainly refers to the passive compliance of employees who are unable to change the existing circumstances. Defensive silence mainly refers to the self-protection of employees to avoid interpersonal alienation from others. Disregardful silence mainly refers to the passive withholding of opinions by employees due to insufficient attachment and identification with the current work or organization. Prosocial silence mainly refers to the silence of employees out of altruistic motives, taking into account the interests of others or the organization and maintaining harmony [6]. Organizational silence in healthcare settings has numerous detrimental effects. For example, it hinders effective communication among healthcare professionals, compromising patient safety and the quality of medical care provided [7, 8]. Furthermore, working in such a negative environment over long periods can significantly affect the job satisfaction and emotional wellbeing of nurses, which can reduce the sense of self-worth and belonging, ultimately increasing turnover rates [9]. Nurses who remain silent in their organization may lose their motivation and ability to learn, which can adversely affect innovation in the hospital setting [10].

Various reasons contribute to the organizational silence of nurses such as managerial style, personal characteristics, and work environment. In terms of age and gender, Harmanci et al. [11] surveyed 601 doctors and nurses in public hospitals regarding organizational silence in the workplace and found that younger nurses tended to conform to others' opinions and were more reluctant to express their own, fearing isolation and disagreement with colleagues, thus leading to a higher incidence of organizational silence. Male nurses experience a greater degree of organizational silence due to their apprehensions about interpersonal relationships [12]. Nurses with extensive work experience, better decision-making abilities, and greater proficiency in utilizing resources to stay up to date with new technologies and advancements in the field of nursing experience less organizational silence [13]. According to most researchers [14–16], as the ability with nurses increases, the degree of organizational silence decreases. This is related to the stronger professional consciousness of nurses with higher qualifications and educational backgrounds. Concerning the employment status, Zhang Shaoguo [17], Cao Rui [14], and other researchers have found that organizational silence is lower among regular staff than temporary staff. This occurs because temporary staff often perceive their jobs to be unstable, which discourages these individuals from being involved and enthusiastic about hospital development. Based on these findings, the first hypothesis (H1) proposed in this study stated that age, gender, undertaking nursing management tasks, professional title, highest education level, and employment status positively affect the organizational silence of nurses.

In terms of leadership style, Morrison [18] argued that the leadership style of managers is the main reason for organizational silence. Leadership styles often possess distinctly individual characteristics. A multifaceted leadership style is a novel method [19] to evaluate human resources and primarily includes transformational and transactional approaches [20]. Transactional leadership is one of the most common leadership behaviors, which exchanges benefits after the workers achieve the set goals [21]. Transformational leadership can make staff realize the importance of work contents, stimulate the higher level needs of employees, establish a harmonious atmosphere between leaders and employees, and motivate workers to pay more attention to the collective interests to achieve the expected results [22]. Similarly, Caylak and other experts [18] stated that in healthcare settings, silence among nurses is mainly caused by management factors. Nurse managers are the leaders closest to frontline nursing staff in hospitals, and they serve as administrators and organizers within the nursing team. According to Detert and Burris [14], a key factor influencing the willingness of employees to speak up is the openness of leadership. When leaders are open-minded and employees are aware of the friendly characteristics of their leadership, the courage to voice different opinions will break the silence of employees in the organization [23]. Some studies have shown that inclusive leadership behavior can reduce the occurrence of silence behavior, while toxic leadership behavior will increase it [24, 25]. Their leadership styles can considerably affect the organizational silence of nurses. Transformational and transactional leadership styles were negatively associated with organizational silence among nurses in China, meaning that the leadership styles mentioned above reduced the occurrence of silent behaviors [26]. This finding has been confirmed in studies in other countries [27]. These studies suggested that leadership style significantly affects organizational silence. Therefore, the second hypothesis (H2) proposed in this study stated that the transformational leadership style of nurse managers can reduce the occurrence of organizational silence of nurses, and the third hypothesis (H3) stated that the transactional leadership style of nurse managers can reduce the occurrence of organizational silence of nurses.

The Multiple Indicators Multiple Causes (MIMIC) model is a type of structural equation modeling, which has a wide range of applications [28], such as in depression among the elderly in community settings [29] and to assess mental health among migrants in urban [30]. It uses manifest variables as explanatory variables, while the explained variables are latent ones that are defined or measured by multiple reflective indicators. In the model constructed in this study, the various dimensions within the “leadership style” scale served as the manifest variables, whereas the explained variable “organizational silence of nurses” was considered to be a latent variable. Unlike the binary logistic model, the MIMIC model caters to situations where explained variables cannot be directly measured, and it provides a more comprehensive assessment of the effect of leadership style on the organizational silence of nurses.

To summarize, the organizational silence of nurses adversely affects medical care, patient safety, and the development of innovative techniques in hospitals. Transactional and transformational leadership styles negatively affect the organizational silence of nurses. Hence, this study was conducted to determine the effect of diverse leadership styles of Chinese head nurses on the organizational silence of nurses and investigate how general demographic information is related to the organizational silence of nurses.

## 2. Methods

### 2.1. Design

In this study, a cross-sectional survey research design was implemented.

### 2.2. Participants

By conducting convenient sampling, clinical nurses were selected from four hospitals in ShenZhen, a city in Guangdong Province, China, and a special economic zone, with 144 hospitals in the city, as research participants from April 2020 to May 2020. The inclusion criteria were as follows: (1) licensed and practicing clinical nurses; (2) those who had previously worked with their head nurses in the same department for at least six months; and (3) those who provided informed consent and volunteered to participate in the study. The exclusion criteria were as follows: (1) clinical nurses on probation, undergoing standardized training, or rotating through departments and (2) those on leave, studying, or away from the hospital for training.

### 2.3. Data Collection

After explaining the purpose of the survey to hospital administrators and obtaining support from the nursing department of the hospital, the researchers distributed an online questionnaire using the Wenjuanxing platform. The online questionnaire was set up with a description at the beginning of the questionnaire explaining the significance, purpose, content, and methodology of the survey. All nurses were informed that participation in the study was voluntary. They could withdraw at any time in the process of filling out the questionnaire. Before the questionnaire was submitted, a message box appeared to inform the nurses whether to confirm the submission or not. If nurses submitted the questionnaire, it demonstrated that they were willing to participate. Second, the online questionnaire did not collect personal information, which could ensure the participants' anonymity. The research team can remove the submitted data if the respondents request to withdraw from the study after submitting the questionnaire, based on the time of submission, IP address, and demographics of the subject. After excluding invalid questionnaires with missing or incomplete responses, a total of 545 valid questionnaires were returned.

### 2.4. Measurements

#### 2.4.1. Demographics

Based on the research objectives and content of this study, the researchers designed a survey to collect data on demographic characteristics, including age, gender, undertaking nursing management tasks, professional title, education level, and employment status of the nurses.

#### 2.4.2. Leadership Style of the Head Nurse

The Manager Multifaceted Leadership Questionnaire was developed by Bass and Avolio [31, 32] and modified into a Chinese version by Hui [33], which was implemented in this study. The questionnaire had two dimensions, including transformational leadership (20 items) and transactional leadership (12 items), with 32 items in total. A higher factor score for each dimension was associated with a higher frequency of a leadership style exhibited. The questionnaire used a five-point Likert scale, with a Cronbach's alpha value of 0.88. All items were positively scored, indicating that higher scores in each dimension reflected a stronger perception of the leadership style of the nurse manager among nurses.

#### 2.4.3. Organizational Silence Among Nurses

The Nurses' Organizational Silence Assessment Questionnaire, adapted by the Chinese scholar Yang Jing [34], comprises four dimensions, including disregardful silence, defensive silence, prosocial silence, and acquiescent silence, with 20 items in total. This scale uses a five-point Likert rating scale, where 1 represents “never felt” and 5 represents “always felt.” The overall Cronbach's alpha coefficient for the questionnaire was 0.92, and Cronbach's alpha coefficients for each dimension ranged from 0.79 to 0.86.

### 2.5. Data Analysis

#### 2.5.1. Descriptive Statistics

All data were analyzed using the SPSS 26.0 software. The scores of the participants (Nurse Manager Multifaceted Leadership Questionnaire and Nurses' Organizational Silence Assessment Questionnaire) were found to be approximately normally distributed (skewness less than 2 and kurtosis less than 5). Descriptive statistics (mean ± standard deviation) were used to summarize the data.

#### 2.5.2. Correlation Analysis

Pearson correlation analysis was conducted to examine the relationship between the transformational leadership style, transactional leadership style, and organizational silence of nurses.

#### 2.5.3. MICIC Model

The initial path diagram of the MICIC model was created in Amos 24, where e1-e4 represented the errors of the manifest variables and e5 represented the error of the latent variable. After running the output calculations in Amos 24, the Model Fit displayed the fitted model. The overall fit of the model to the data was evaluated using several evaluation metrics. The following criteria were used to assess the model: *χ*^2^/df < 3 indicated an excellent fit, *χ*^2^/df < 5 indicated an acceptable fit, RMSEA < 0.05 indicated a good fit, and RMSEA < 0.08 indicated a reasonable fit. These criteria may not be strictly applied when the sample size is large. If CFI, TLI, and IFI are greater than 0.9, a good model fit is implied [35]. The significance level was set at *p* < 0.05. By examining the relationships between transactional and transformational leadership styles and evaluating the relationships between residuals, the model was refined. Finally, the revised model path diagram was obtained.

### 2.6. Ethic Consideration

The Ethics Committee of Fuwai Central China Cardiovascular Hospital excused the study from ethical assessment. The subjects gave informed consent after being told about the goal of the study. The ability to exit the study at any time has been ensured.

## 3. Results

### 3.1. Sample Characteristics and Univariate Analysis

In this study, most respondents were female (96.3%); the largest age group was 21–30 years old, accounting for 47.5% of the total sample. Among the participants, 81.1% had not undertaking nursing management tasks, whereas 62.0% were primary nurses. The predominant educational background was a bachelor's degree (76.7%), and there were more temporary staff (77.6%) than regular staff (22.4%). The results of one-way ANOVA showed that the differences in the organizational silence of nurses were not significant when comparing different ages, genders, professional titles, and undertake nursing management tasks (*p* > 0.05). However, the difference in terms of employment status was significant (*p*=0.036) (see Table 1 for details).

### 3.2. Transformational Leadership Style, Transactional Leadership Style, and Organizational Silence of Nurses

Among the multiple leadership styles of head nurses, the score for transformational leadership (42.16 ± 15.47) was higher than that for transactional leadership (31.01 ± 5.69). The overall score for the organizational silence of the nurses was 47.71 ± 16.44. Among the various dimensions, the scores for prosocial silence, acquiescent silence, and defensive silence were 10.19 ± 3.84, 7.69 ± 3.18, and 14.61 ± 5.19, respectively; the highest score was for disregardful silence (15.21 ± 5.46).

### 3.3. The Correlation Between the Transformational Leadership Style, Transactional Leadership Style, and Organizational Silence of Nurses

The results of the correlation analysis between the transformational leadership style, transactional leadership style, and organizational silence of nurses are presented in Table 2. The transformational leadership style and transactional leadership style were positively correlated (*r* = 0.681, *p* < 0.01); moreover, the transformational leadership style and organizational silence of nurses were positively correlated (*r* = 0.497, *p* < 0.01). In addition, the transactional leadership style was positively correlated with the organizational silence of nurses (*r* = 0.301, *p* < 0.01).

### 3.4. Results of the MICIC Model

**Figure 1:**
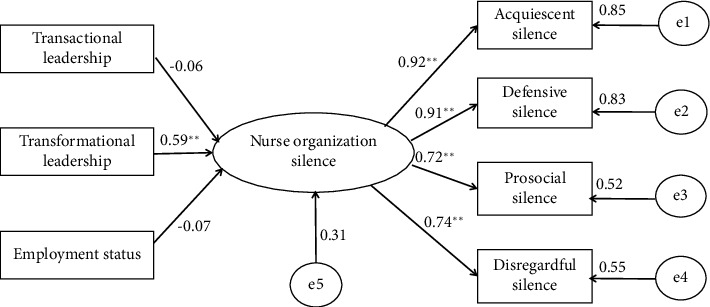
Nurses' organizational silence MICIC model estimation results (standardized coefficients). Note: All numbers are standardized loadings/coefficients. ^∗∗^*p* < 0.001.

The estimation results of standardized coefficients in the MICIC model are shown in Figure 1. The final model had acceptable fit indices (χ^2^/df = 4.07 < 5; CFI = 0.983 > 0.9; TLI = 0.969; IFI = 0.983 > 0.9; and RMSEA = 0.075 < 0.08). Among the basic demographic variables, the employment status held negatively affected the organizational silence of nurses, although the effect was not significant (*β* = −0.07, *p* > 0.05). Among the multiple leadership styles, only transformational leadership had a significant positive effect on the organizational silence of nurses (*β* = 0.59, *p* < 0.001). Transactional leadership negatively affected the organizational silence of nurses, although the effect was not significant (*β* = −0.06, *p* > 0.05). Within the organizational silence of nurses, acquiescent silence had the strongest positive effect (*β* = 0.92, all *p* < 0.001).

## 4. Discussion

The results of this study indicated that the overall score for organizational silence among nurses was 47.71 ± 16.44, which was at a moderately low level and might occur because of several reasons: (1) nurses face heavy workloads and significant stress, which often leaves these individuals with little time and energy to suggest improvements. This can decrease their feeling of involvement. (2) Leadership and management styles also play a role. Traditional authoritarian leadership can make nurses feel that their opinions are unimportant or unused. In an unfair work environment, employees may choose not to share their views [36]. (3) Personal factors such as personality traits, lack of experience, and confidence can also lead nurses to withhold their opinions. (4) Although an open and inclusive atmosphere in an organization encourages nurses to express their ideas, nurses may be silenced by concerns about the interests of the organization, thus ensuring the sense of belonging to the organization [37]. Among the four dimensions of organizational silence, the scores, from highest to lowest, were as follows: disregardful silence (15.21 ± 5.46), defensive silence (14.61 ± 5.91), prosocial silence (10.19 ± 3.84), and acquiescent silence (7.69 ± 3.18). These results were similar to those reported by Meng Wei [38]. Disregardful silence mainly refers to the passive withholding of opinions by employees due to insufficient attachment and identification with the current work or organization. The largest proportion of participants in this study (approximately 47.5%) were between 21 and 30 years old. Younger nurses, with less work experience and seniority, may contribute to higher levels of disregardful silence.

The results of the univariate analysis showed that the differences in scores among nurses varying in age, gender, professional title, undertake nursing management tasks, and the highest education level were not significant (*p* > 0.05), which was different from the findings [36]. This finding suggested that these variables were not the main factors influencing the organizational silence of the nurses in this study. The reasons may be as follows: (1) the participants may share similar demographic characteristics. For example, in this study, the nurses were predominantly female (96.3%) who were 21–30 years old (47.5%), mostly primary nurse (60%), and not undertaking nursing management tasks(81.1%). Therefore, the differences in these characteristics were not significant. (2) Insufficient precision or accuracy in the definition of variables might have led to failure to detect individual differences. (3) Univariate analysis has certain limitations because it only considers the relationship between one variable and the dependent variable, disregarding other possible factors. Thus, individual effects of variables such as age and gender might be masked by how they interact with each other, leading to inconclusive results. However, the differences in scores among nurses with different employment status were found to be significant (*p* < 0.05), which was consistent with the findings [12]. The reasons for this may be as follows: (1) nurses with different employment status have different experiences, attitudes, and feelings regarding their work in the hospital, which can result in differences in the occurrence of organizational silence. (2) Regular and temporary nurses differ in their emotional attachment to their work. Regular nurses are more sensitive to existing issues and are more willing to express their opinions. (3) As regular nurses have greater opportunities for promotion and are more valued by managers, they are more likely to be more actively involved in hospital development [39]. In contrast, as temporary nurses have lower job stability, they tend to maintain a neutral attitude toward organizational issues. Therefore, managers should provide training and promotion opportunities to temporary nurses, develop strategies to give rewards, praise individuals, and provide allowances for exceptional performance to encourage their active engagement in work.

Second, the results of the correlation analysis showed that the transformational leadership style and organizational silence among nurses were positively correlated (*r* = 0.497, *p* < 0.01). This finding indicated that the higher transformational leadership style exhibited by head nurses, the higher organizational silence among nurses. Kark et al. [40] found that although transformational leaders possess strong abilities and can effectively assign tasks and provide assistance to their workers, over time, they may become dependent on their managers. When addressing technical and managerial issues in their work, nurses who lack self-reflection skills may struggle to articulate their exploratory ideas. Transformational leaders focus on inspiring their workers and encouraging them to exceed their limitations. However, this approach can sometimes make nurses feel apprehensive about possible failures, causing these individuals to remain silent when confronted with issues. Furthermore, the transactional leadership style and organizational silence among nurses were also positively correlated (*r* = 0.301, *p* < 0.01), which indicated that as the level of transactional leadership style increased among head nurses, the level of organizational silence among nurses also increased. Chen [41] suggested that the contingency management characteristics of transactional leadership can negatively affect the job performance and satisfaction of individuals. A decline in the job satisfaction and engagement of nurses can also contribute to the occurrence of organizational silence. Garon et al. [42] emphasized the importance of an open communication environment in encouraging nurses to voluntarily share their ideas with managers. Managers must cultivate an inclusive work environment that empowers nurses, gives individuals opportunities to make decisions and reflect, and provides support and training to boost their confidence and resilience.

Finally, analysis of the MICIC model revealed that transformational leadership was the sole significant predictor of the organizational silence of the nurses (*β* = 0.59, *p* < 0.001). However, H2 was not supported, which means that transformational leadership cannot reduce the occurrence of organizational silence among nurses. It is possible that nurses may hesitate to seek feedback from their superiors because of embarrassment or fear of harming their professional reputation [43]. If nurses perceive seeking feedback or other important information from transformational leaders to be less beneficial, they may become less likely to communicate. The MICIC model showed that transactional leadership style and employment status adversely affected the organizational silence of nurses. Specifically, both variables had a slight negative impact (*β* = −0.06 and *β* = −0.07); however, these effects were not statistically significant (*p* > 0.05 for both). Thus, H1 and H3 were not supported. The results were not significant, probably because of several factors. First, the small effect size (*β* = −0.07, *β* = −0.06) may have contributed to the lack of statistical significance, even if a relationship between the variables was present [35]. Second, individual differences among nurses in their perceptions and reactions toward employment status and transactional leadership styles may have led to varying results. Finally, the relationship between employment status and the organizational silence of nurses may be complex and influenced by other variables, such as nonlinear relationships or interaction effects. Although the results of the MICIC model did not show a significant relationship, employment status and transactional leadership styles may have some effect on the organizational silence of nurses.

### 4.1. Implications for Nursing Management

Organizational silence among nurses is a common issue in clinical settings. It is affected by employment status, transactional leadership, and transformational leadership. To address this problem, managers can consider the following steps. Transformational leadership styles can inspire a sense of mission and achievement motivation among nurses and may also inadvertently impose pressure on them to express themselves, in order to ensure the sense of belonging to the organization. Consequently, nursing managers should timely adjust their leadership styles to help nurses establish psychological safety [44], ensuring that nurses feel they will not be punished for expressing uncertainty or experiences of failure, thus alleviating their concerns [45]. Moreover, there are differences in how nurses accept various leadership styles. Some nurses may prefer a transactional leadership style with clear structure and explicit guidance. Leaders should pay attention to individual differences and adjust their leadership style according to the preferences and needs of nurses, achieving personalized adaptation of leadership. Nursing managers should focus on the importance of organizational support [46], and reward nurses who speak up create a culture where voicing opinions is valued and encouraged. Finally, special attention should be given to temporary nurses. Nursing managers should provide skill-based training, enhance their professional value, and improve their job satisfaction. Listening to their ideas, and fostering a sense of teamwork can encourage temporary nurses to speak up [47]. Adopting an open, respectful, and supportive leadership style may help nurses feel more confident in voicing their concerns. This, in turn, may improve patient care and safety.

### 4.2. Limitations and Recommendations

Our study had some limitations. First, it was a cross-sectional study, and thus, inferences regarding cause-and-effect relationships between variables cannot be drawn. Second, questionnaires were only collected from nurses in hospitals in Shenzhen. And although nursing has long been a female-dominant profession in terms of its demographical profile in China [48], the sample is the large female representation in our study. Thus, the results might not apply to other areas or groups. Finally, the data were collected based on the memories and opinions of nurses, which might have introduced biases in the results. Even with these limitations, this study was unique. The MICIC model was used to combine factor and regression analyses. It provided insights into how different leadership styles and employment status affect the silence of nurses in organizations. The results showed that transformational leadership can aggravate this organizational silence. Based on these findings, practical advice was provided for clinical settings.

## 5. Conclusion

Through a cross-sectional survey of clinical nurses using the MICIC model, this study concluded that the organizational silence of the nurses was at a moderately low level. The employment status was identified as a key factor influencing the organizational silence of nurses, while transformational leadership style can contribute to the organizational silence of nurses. Therefore, nursing managers should cultivate a sincere, open leadership style, pay attention to temporary nurses, and individual differences and adjust their leadership style according to the preferences and needs of nurses, achieving personalized adaptation of leadership.

## Figures and Tables

**Table 1:** Differences in organizational silence among nurses with different demographics (*N* = 545).

	Group	Numbers (%)	$\overset{¯}{x}\pm s$	*F*	*p*
Age	21–30	259 (47.5)	47.79 ± 17.20	0.146	0.932
	31–40	210 (38.5)	47.27 ± 15.40		
	41–50	64 (11.7)	48.52 ± 17.40		
	51–60	12 (2.2)	49.42 ± 13.04		

Gender	Female	525 (96.3)	47.80 ± 16.27	0.464	0.496
	Male	20 (3.67)	45.25 ± 20.85		

Professional titles	Primary nurse	338 (62.0)	47.04 ± 17.10	0.921	0.399
	Nurse-in-charge	174 (31.9)	49.10 ± 15.72		
	Chief superintendent nurse	33 (6.05)	47.18 ± 12.86		

Undertake nursing management tasks	No	442 (81.1)	47.53 ± 16.92	0.269	0.604
	Yes	103 (18.9)	48.47 ± 14.25		

Education level	Associate's degree and below	119 (21.5)	48.16 ± 16.84	1.751	0.175
	Bachelor's degree	418 (76.7)	47.78 ± 16.38		
	Master's degree and above	8 (1.5)	37.00 ± 10.72		

Employment status	Regular workers	122 (22.4)	50.46 ± 16.80	4.429	0.036
	Temporary workers	423 (77.6)	46.91 ± 16.27		

**Table 2:** Correlation analysis between nurses' organizational silence and nurse leaders' transactional and transformational leadership styles (*n* = 545).

Dimension	Transactional style	Transformational style	Prosocial silence	Disregardful silence	Acquiescent silence	Defensive silence	Organizational silence
Transactional style	1						
Transformational style	0.681^∗∗^	1					
Prosocial silence	0.201^∗∗^	0.333^∗∗^	1				
Disregardful silence	0.193^∗∗^	0.366^∗∗^	0.562^∗∗^	1			
Acquiescent silence	0.351^∗∗^	0.562^∗∗^	0.666^∗∗^	0.662^∗∗^	1		
Defensive silence	0.278^∗∗^	0.449^∗∗^	0.782^∗∗^	0.705^∗∗^	0.839^∗∗^	1	
Organizational silence	0.301^∗∗^	0.497^∗∗^	0.844^∗∗^	0.798^∗∗^	0.917^∗∗^	0.957^∗∗^	1

^∗∗^ *p* < 0.01.

## Data Availability

The data that support the findings of this study are available from the corresponding author upon reasonable request.
